# Effects of phthalimidoperoxycaproic acid (PAP+) associated or not with LED irradiation as a bleaching agent: an in vitro study

**DOI:** 10.1007/s10103-026-04888-3

**Published:** 2026-05-20

**Authors:** Samuel da Silva Palandi, Marcos Roberto de Lima Benati, Beatriz de Cássia Romano, Matheus Kury, Vanessa Cavalli

**Affiliations:** 1 Department of Restorative Dentistry, State University of Campinas, Campinas, Brazil; 2 Graduate Program in Dentistry, Paulista University (UNIP), São Paulo, Brazil

**Keywords:** Dental bleaching, Hydrogen peroxide, Tooth bleaching agents, Light-emitting diodes, Over-the-counter

## Abstract

Objective: To evaluate the efficacy and safety of phthalimidoperoxycaproic acid (PAP+) as a bleaching agent, combined or not with blue or violet LEDs. Materials and methods: 108 sound bovine enamel/dentin blocks stained with black tea were assigned to nine groups (n = 12): Control (no treatment - NC); MMO (Bright max violet LED, MMOptics); VIO (VIO405, HiSmile); PAP (PAP+ - VIO405 whitening kit, HiSmile); PAP/MMO; PAP/VIO; HP10 (10% Hydrogen Peroxide - White Class 10%, FGM); HP10/MMO; HP10/VIO. LED irradiation and light potency were analyzed, and the pH of the gels was assessed before and after treatment. Afterwards, dental blocks underwent treatment for 7 days according to each protocol. Samples were assessed for color change (ΔE_00_), whiteness index (ΔWI_D_), microhardness analysis and enamel morphology (SEM). Data were analyzed by two-way ANOVA and Tukey or Bonferroni post-hoc test (α = 0.05), and Linear Mixed Models and Tukey-Kramer (α = 0.05). Results: VIO delivered approximately 10% of MMO irradiance. Irradiation reduced gel pH, but values remained above 5.8. PAP did not differ from controls, regardless of LED use (p > 0.05), whereas HP10 showed greater bleaching efficacy, especially when combined with MMO (p < 0.05). No morphological enamel damage was observed, and microhardness increased over time in all groups (p < 0.05). Conclusion: VIO exhibited lower power and stability than MMO, but arguably, both enhanced the efficacy of HP10. Under the tested protocol, PAP+ failed to induce significant bleaching, regardless of association to LED irradiation, while HP10 effectively induced bleaching independently of LED association. Importantly, none of the tested treatments resulted in microhardness loss or morphological damage to the enamel surface. Clinical relevance: PAP + was not able to promote significant bleaching in the adopted protocol, showing mostly similarities to the NC group, and did not improve the LED devices efficacy. PH10 consistently showed significant bleaching, benefiting from the association with MMO. MMO alone was able to produce light bleaching effects.

## Introduction

Dental bleaching has become one of the most frequently performed esthetic procedures in modern dentistry, reflecting the growing emphasis on smile harmony and the continuous development of esthetic dental technologies [1]. The widespread adoption of safe and predictable techniques has substantially increased patient access to bleaching treatments, both in-office and in supervised at-home modalities [2]. However, technological advancements and the development of new formulations have enabled the use of lower concentrations of active agents, often combined with adjuvants and light-activation devices, while maintaining bleaching efficacy and minimizing adverse effects [3]. Several parameters directly influence the performance of bleaching agents, including the type of active compound, its concentration and pH, the presence of remineralizing or desensitizing additives, and the potential activation by external energy sources, such as light-emitting diodes (LEDs) or lasers [4–6]. This combination of variables explains the wide diversity of available clinical and at-home protocols, all aiming to optimize bleaching efficacy while preserving the integrity of dental tissues [3].

Hydrogen peroxide remains the gold standard among bleaching agents, used either alone or in the form of carbamide peroxide. Its effect results from diffusion through the interprismatic enamel structure into the dentin, where it oxidizes the unsaturated organic molecules responsible for tooth discoloration, also referred to as chromophores [1]. This reaction, mediated by reactive oxygen species (ROS), cleaves the chromophoric chains into smaller, colorless compounds [2]. The process acts mainly on intrinsic pigments, which are resistant to mechanical removal. Despite its proven efficacy, peroxide use is associated with potential adverse effects. Under acidic conditions, reductions in enamel microhardness, increases in surface roughness, and prism exposure have been reported [4]. The formulation of neutral pH products containing remineralizing agents, such as fluoride or hydroxyapatite, has been shown to effectively minimize these alterations [7, 8]. In soft tissues, direct contact of the bleaching gel may cause gingival irritation, which is why the use of protective barriers or individualized trays is strongly recommended [9].

To reduce adverse effects and improve patient comfort, several strategies have been explored, including the use of low-peroxide concentrations combined with desensitizing agents, the incorporation of nanoparticles, and photochemical activation using LED light [10, 11]. The application of light sources, particularly light-emitting diodes (LEDs), has proven to be an effective adjuvant when combined with low-concentration bleaching gels, resulting in reduced discomfort and a lower risk of hypersensitivity compared to standard protocols using high-concentration peroxide alone [10, 12–14]. The main mechanism proposed involves a mild increase in gel temperature during irradiation, which facilitates hydrogen peroxide dissociation and accelerates the generation of oxidative radicals [5]. This faster reaction limits deeper diffusion of the agent, thus reducing dentin penetration and potentially minimizing post-treatment sensitivity [15]. It is noteworthy that, although to a lesser extent, light irradiation alone may also induce tooth bleaching, possibly due to direct interaction between light and extrinsic chromophores adhered to the outer enamel surface, leading to molecular excitation and fragmentation [5].

Concurrently, increasing regulatory restrictions on the use of peroxides in over-the-counter products have driven research toward alternative agents with improved biocompatibility profiles. Phthalimidoperoxycaproic acid (PAP) has emerged in this context as a promising alternative. PAP is a stable organic peracid whose action does not involve the release of free radicals or ROS but rather involves selective epoxidation reactions targeting chromophore double bonds [16–18]. This mechanism minimizes the risk of damage to dental hard and soft tissues while preserving enamel structure [16, 19]. PAP has been introduced as an alternative bleaching agent in at-home and over-the-counter products. Although it has demonstrated the ability to promote tooth color change in experimental and clinical settings [16–19], the available evidence remains limited when compared to conventional peroxide-based agents.

Overall, studies evaluating PAP-based bleaching protocols have reported heterogeneous results [16–18], and data regarding its interaction with light activation are scarce [19]. While some commercial protocols incorporate LED devices in association with PAP, a synergistic effect between PAP and irradiation on bleaching efficacy and enamel-related outcomes remains unclear. Therefore, the present study aimed to evaluate the effect of PAP in combination with different LED devices on dental bleaching. The null hypotheses tested were: (1) different LED devices used for light activation would not differ in irradiance and power output; (2) PAP would not differ from 10% hydrogen peroxide (HP10) in bleaching efficacy; and (3) PAP would not induce changes in enamel surface properties.

## Materials and methods

### Experimental design

This study was conducted in two phases. In phase 1, the LED devices (MMO - Bright max - MMOptics) and VIO (VIO 405 - HiSmile) were characterized for spectral irradiance and irradiance over time. The pH of the bleaching agents: 10% hydrogen peroxide (White Class – FGM) and Phthalimidoperoxycaproic acid (PAP+ - HiSmile) were assessed before and after application, according to the bleaching protocols (Table 1). In phase 2, bovine enamel/dentin blocks stained with black tea were assigned to nine groups (*n* = 12), and submitted to treatments combining (or not) the bleaching agents tested (HP10% and PAP) with violet LED light (MMO - Bright max - MMOptics) or VIO (VIO 405 - HiSmile), as follows:

- C: Control group (no treatment)
- MMO: irradiation with MMO without gel or acid;
- VIO: irradiation with VIO without gel or acid;
- PAP: phthalimidoperoxycaproic acid (PAP+) treatment;
- HP10: 10% hydrogen peroxide treatment;
- PAP/MMO: PAP treatment combined with MMO irradiation;
- PAP/VIO: PAP treatment combined with VIO irradiation;
- HP10/MMO: HP10 bleaching combined with MMO irradiation;
- HP10/VIO: HP10 bleaching combined with VIO irradiation.

**Table 1:** Basic composition of products, and bleaching protocols according to treatment

**Product**	**Composition**
10% Hydrogen peroxide (White class, FGM) Lot: 021023	10% Hydrogen peroxide, neutralized carbopol, potassium nitrate, sodium fluoride, calcium gluconate, stabilizer, humectant, deionized water.
PAP+ (HiSmile) MFG: 09112021-A	Glycerin, water, phthalimidoperoxycaproic acid (PAP), potassium citrate, ammonia, acrylates/dimethyltauramide VP copolymer, hydroxyapatite, PVP, sodium saccharin, potassium hydroxide, peppermint oil, mica, titanium dioxide.
**Product**	**Protocol of application**
HiSmile PAP+ (PAP)	The gel was applied in a 2 mm-thick layer on the specimen surface for 10 min. After this period, the samples were rinsed with distilled water in order to remove all the gel. This protocol was carried out over seven sessions with daily applications.
10% Hydrogen Peroxide (HP10)	A thin layer (1 mm) of gel was applied across the entire enamel surface and left in place for 30 min. After the exposure time, the blocks were rinsed with distilled water to ensure complete removal of the gel. This protocol was performed for seven days, with daily applications.
MMOptics Bright Max (MMO)	The cleaned samples were positioned 8 mm from the light source and irradiated in four cycles consisting of 5 min of exposure followed by 30 s intervals. This protocol was performed for seven consecutive days, with daily applications.
HiSmile VIO405 light (VIO)	The cleaned samples were positioned 8 mm from the light source and continuously irradiated for 10 min. This protocol was performed for seven consecutive days, with daily applications.
PAP/VIO	All combinations of LEDs (VIO and MMO) and gels (PAP, HP10) were carried out using the same protocol. The cleaned samples received a uniform 1.5 mm layer of gel and were irradiated according to the specifications of each LED. After exposure, the samples were rinsed with distilled water to completely remove the gel. This protocol was performed over seven sessions with daily applications.
PAP/MMO	
HP10/VIO	
HP10/MMO	

The control group was not submitted to any treatments and remained stored in remineralizing solution (1.5 mM Ca; 0.9 mM PO_4_ and 150 mM KCl in a 20 mM tris buffer solution, pH 7.0) throughout the experimental phase. Color (∆E_00_) and whiteness index (∆WI_D_) changes were evaluated at T_0_ (after staining), T_1_ (24 h after treatment), and T_2_ (14 days after treatment). At T_2_, three samples from each group were selected randomly and the surface morphology was observed under scanning electron microscopy (SEM). Data were analyzed according to the specific statistical test adopting a significance level of 5%.

### Sample size calculation

The sample size calculation was based on the study by Muller-Heupt et al. [18], in which the authors evaluated dental color change (ΔE) promoted by PAP compared to other at-home treatments and over-the-counter products. Based on the primary outcome (ΔE) reported in that study (*n* = 10), the means and standard deviations of the PAP group (ΔE = 6.6 ± 3.3) and the untreated control group (ΔE = 2.5 ± 1.3) were used to calculate the sample size, adopting a significance level of 5% and a power test of 80% (1 − β). Under these conditions, the results indicated that 11 specimens per group would be required (*n* = 11). Considering the lack of additional reports on PAP efficacy and the relatively high coefficients of variation reported by Muller-Heupt et al. [18] (50% for PAP and 26% for the control group), the final sample size was increased to 12 specimens per group (*n* = 12).

### 1° Phase: characterization of LED devices and pH of gels

#### Determination of spectral irradiance and irradiance over-time

**Fig. 1:**
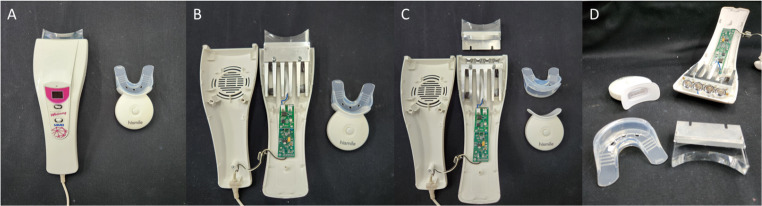
**A**: LED devices with barrier attached; **B**: Opening of MMO device to expose the screws for barrier disassembly; **C**: Removal of barriers (Acrylic tip of MMO and silicone tray of VIO).; **D**: Close-up of barriers and arrangement of LED on the devices

To characterize the LEDs, a spectrometer (MSC15W, SN 37560; Gigahertz-Optik, Amesbury, MA, USA) and a specific software (MSC15 measurement software v.2019.1.0; Gigahertz-Optik, Amesbury, MA, USA) were used. The integrating sphere was consistently positioned at a distance of 8 mm from the central LED of the devices, simulating interaction with a central incisor [20]. The cycles recommended by the manufacturers were performed to determine the irradiance and power output of the devices. Due to device differences, VIO measurements were taken both with and without its silicone tray-like barrier. For the MMO device, measurements were performed with its acrylic light-transmitting tip and after its removal (Fig. 1A-D).

#### pH analysis of the bleaching agents

The pH of the bleaching agents was determined using a pH meter (Equilam, Diadema, SP, Brazil) coupled to a potentiometer (Orion Research Inc., Boston, MA), calibrated with pH 4.0 and 7.0 buffer solutions. Following the methodology of Palandi et al. [21], gel samples were diluted in distilled water at a 1:10 ratio, and the pH was measured by fully immersing the microelectrode into the resulting solution. Measurements were performed in triplicate at baseline (0 min) and upon completion of each treatment: 10 min (VIO, VIO/PAP, VIO/HP10, PAP), 22 min (MMO, MMO/PAP, MMO/HP10), and 30 min (HP10).

### 2° Phase: color, enamel hardness and morphology

#### Preparation of the specimens

A total of 300 bovine incisors were collected and stored in 0.1% thymol solution at 4 °C. The crowns were cleaned and debris removed using periodontal curettes. Then, crowns were separated from the roots with a diamond disc mounted on a handpiece, 2 to 3 mm apical to the cementoenamel junction. Blocks measuring 5 mm × 5 mm were obtained by making mesiodistal and cervico-incisal cuts from the coronal region, also using a handpiece with a mounted disc.

The blocks were fixed onto acrylic stubs with the enamel surface facing the stub, to allow dentin surface planification using aluminum oxide abrasive papers (grit #600) on a rotary polishing machine (Arotec Ind. Com., São Paulo, Brazil). A digital caliper was used to allow dentin thickness of 2 mm. After dentin flattening, the blocks were repositioned with the enamel surface facing upwards, being minimally abraded using aluminum oxide abrasive papers (grit #600), achieving a minimum enamel thickness of 1 mm. Subsequently, the enamel surface was polished with #1200 and #2000 grit paper, followed by polishing with diamond pastes of 6 μm, 3 μm, 1 μm, and ¼ µm granulations. In total, 350 enamel/dentin blocks were properly polished and evaluated for enamel integrity, with only those free from cracks or defects being selected for initial analyses.

#### Staining with black tea

Prior to analysis, the specimens were stained with black tea. The dentin surface was isolated with sticky wax, exposing only the enamel surface. The specimens were immersed in a black tea solution prepared as follows: 2 g of black tea were infused in 100 mL of distilled water for 5 min. The solution was then filtered and buffered to a neutral pH (7.0) using sodium hydroxide (NaOH). Specimens were kept in the solution for 4 h under continuous agitation at room temperature. To stabilize the color before treatments, specimens were stored in a remineralizing solution (1.5 mM Ca, 0.9 mM PO4, 150 mM KCl in 20 mM TRIS buffer, pH 7.0) at 37 °C for 7 days, with the solution being replaced every 48 h [21].

#### Treatments

Table 1 summarizes the chemical composition of the bleaching agents and their respective application protocols. The protocols followed as closely as possible the manufacturers’ instructions, ensuring experimental standardization and comparability among the tested groups. Among treatment sessions and during the 14-day post-treatment period, specimens were stored in the remineralizing solution described above at 37 °C. The solution was refreshed every three days to ensure stable mineral concentrations.

#### Microhardness analysis

Enamel surface microhardness was assessed by performing 3 indentations on the central region of each specimen using a Knoop indenter (Future Tech-FM-1e, Tokyo, Japan), under a static load of 50 g for 5 s, with a spacing of 100 μm between indentations. Microhardness measurements were obtained at T_0_ (after staining), T_1_ (24 h after bleaching), and T_2_ (14 days after bleaching), and the values were converted into Knoop Hardness Number (KHN).

#### Colorimetric analysis

Color changes were evaluated using a digital spectrophotometer (EasyShade V, Vita Zahnfabrik, Bad Säckingen, Germany). The device was stabilized so that the tip remained in constant contact with the enamel surface. Specimens were positioned on the surface of an opaque white ceramic tile, with the dentin side facing the tile. The entire setup was placed inside a light chamber with standardized illumination (GTI, Micrometer). For each specimen, three measurements were performed, rotating the disk 45° between measurements, and the average was calculated. Color parameters (L*, a*, b*, C*, h*) were assessed at T_0_, T_1_, and T_2_. The L* coordinate represented lightness (0 = black to 100 = white); a* and b* represented the chromaticity from green to red and from blue to yellow, respectively; C* represented chroma (color saturation), and h* the hue angle. After recording the L*, a*, b*, C*, and h* values, the color difference was calculated using the CIEDE 2000 formula (ΔE_00_), with ΔE_00_1 = T_1_ − T_0_ (24 h post-treatment) and ΔE_00_2 = T_2_ − T_1_ (14 days post-treatment). In addition to ΔE_00_, the Whiteness Index for Dentistry (WI_D_) was calculated using the parameters L*, a*, and b*. The change in the whitening index was calculated using the formula: ∆WI_D_ = 0.511 L − 2.3424a − 1.100b [22], where higher WI_D_ values indicate whiter specimens and lower values indicate darker specimens. The difference in WI_D_ before and after treatment was expressed as: ΔWI_D_ = WI_D_ (T_1_, T_2_) − WI_D_ (T_0_). ΔE_00_ values adopted for perception (PT) and acceptance (AT) limits (50:50%) were 0.8 (PT) and 1.8 (AT) units, respectively. ΔWI_D_ adopted for PT and AT limits (50:50%) were 0.72 (PT) and 2.62 (AT) units, respectively [23, 24].

#### Scanning electron microscopy (SEM)

Fourteen days elapsed from bleaching (T_2_), three specimens per group were randomly selected for SEM analysis (JEOL-JSM, 6460LV, Tokyo, Japan). The enamel blocks were mounted on aluminum stubs with conductive carbon tape and sputter-coated with a thin gold layer (Balzers-SCD 050, Liechtenstein). Representative qualitative images of the enamel surface morphology were then acquired at magnifications ranging from 1,000× to 3,000×.

#### Statistical analysis

The data were processed by a blinded operator to ensure no bias using SPSS-23 software (IBM-USA). The data were tested for normality and homoscedasticity using the Shapiro-Wilk and Levene tests, respectively. pH data were tested by Two-way ANOVA with repeated measurement and pos hoc of Bonferroni, color parameters (∆E_00_ and ∆WI_D_) were tested by two-way ANOVA and Tukey test (α = 0.05). Microhardness was assessed by Linear Mixed Models and Tukey-Kramer (α = 0.05).

## Results

### LED characterization

**Fig. 2:**
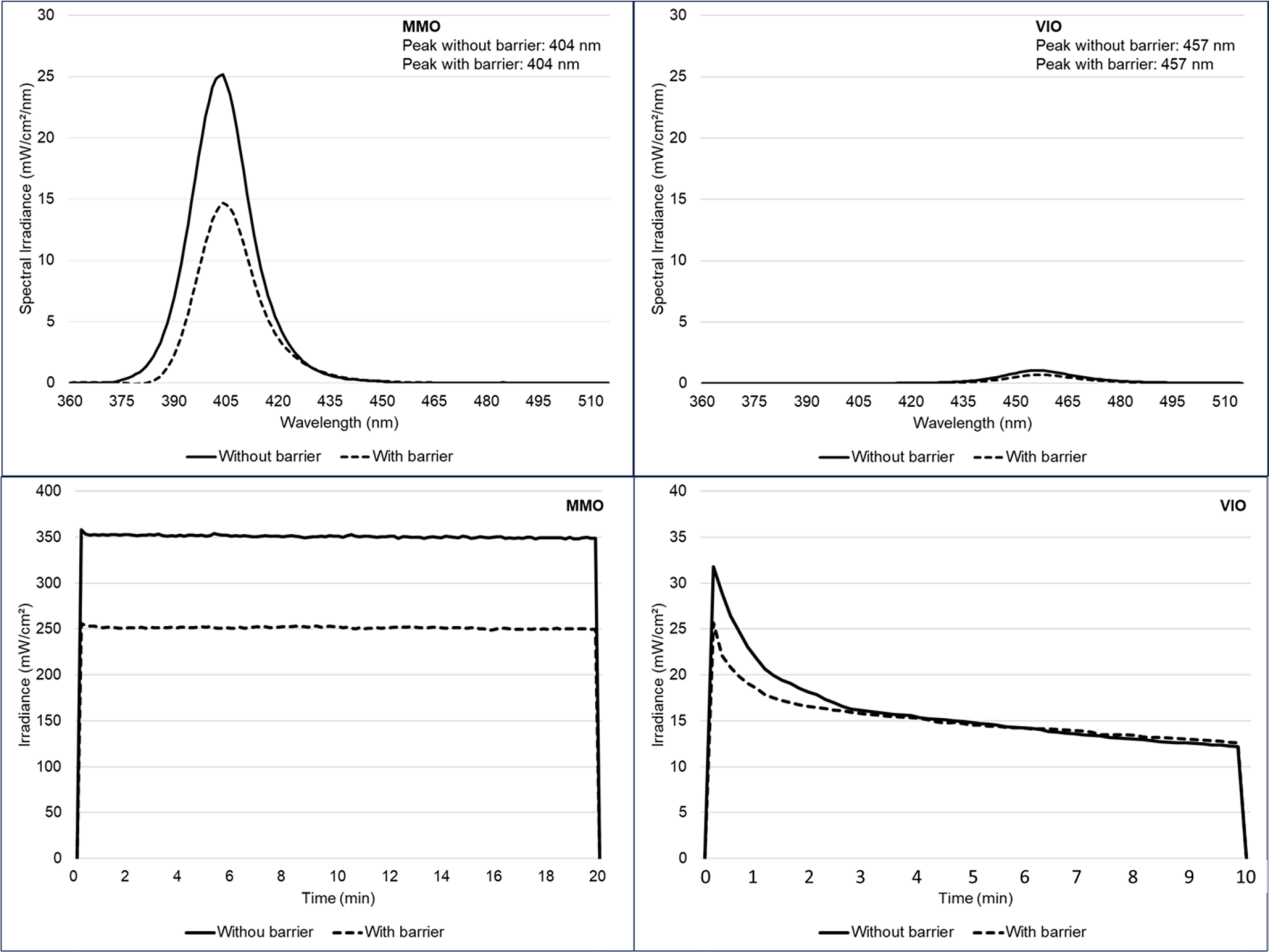
Irradiance spectrum and irradiance over time according to device and condition during data collection (with and without barrier)

Figure 2 illustrates the spectral irradiance of the devices. Emission peaks remained stable regardless of the barrier, with MMO peaking at 404 nm (violet) and VIO at 457 nm (blue). The use of the device’s barrier reduced irradiance in both MMO and VIO. For MMO, irradiance decreased from 350 to 250 mW/cm² but remained stable over time. In contrast, VIO exhibited a time-dependent reduction, decreasing from approximately 30 (without barrier) and 25 mW/cm² (with barrier) to about 12 mW/cm² after 10 min.

### pH analysis of the bleaching agents

The pH values of the gels before and after treatment are presented in Table 2. At the initial time point, similar pH values were observed within the specific products. No statistically significant differences were detected among the groups treated with HP10 (*p* > 0.05), nor among those treated with PAP (*p* > 0.05). However, when comparing HP10-based and PAP-based protocols, statistically significant differences were observed between these treatments (*p* < 0.05), indicating a more acidic initial profile associated with PAP.

**Table 2:** Mean and standard deviation of pH of gels combined or not to the LED devices, before and after the treatments

Treatment	Initial pH	Final pH
HP10	6.08 (0.08) Ab	6.17 (0.06) Aa
HP10/MMO	6.09 (0.04) Aa	5.92 (0.04) Bb
HP10/VIO	6.10 (0.04) Aa	6.03 (0.06) BCa
PAP	5.84 (0.04) Bb	5.98 (0.08) BCa
PAP/MMO	5.86 (0.03) Ba	5.80 (0.05) Da
PAP/VIO	5.86 (0.06) Ba	5.81 (0.03) Da

Treatment times: 10 min (VIO, VIO/PAP, VIO/HP10, PAP), 22 min (MMO, MMO/PAP, MMO/HP10), and 30 min (HP10). Uppercase letters compare treatments at the same time (columns). Lowercase letters compare times within the same treatment (rows)

According to Two-Way ANOVA with pos-hoc of Bonferroni (*p* ≤ 0.05)

This initial homogeneity was disrupted at the final evaluation. HP10 applied alone exhibited the highest pH values and differed statistically from all other groups (*p* < 0.05). When HP10 was combined with MMO or VIO irradiation, the resulting pH values were similar to those observed for the PAP-only group (*p* > 0.05). Nevertheless, HP10/MMO showed a statistically significant difference when compared with HP10/VIO (*p* < 0.05).

The groups treated with PAP associated with MMO or VIO irradiation showed similar pH values to each other (*p* > 0.05), but differed significantly from all other experimental groups (*p* < 0.05), presenting the lowest pH values at the final time point.

Regarding the comparison between initial and final time points, no statistically significant differences were observed for the HP10/VIO, PAP/MMO, and PAP/VIO groups (*p* > 0.05). The HP10 and PAP groups without light activation exhibited a significant increase in pH over time (*p* < 0.05), whereas HP10/MMO demonstrated a significant decrease (*p* < 0.05). Regardless of the combination of factors, all groups presented pH values above 5.8.

## Colorimetric analysis

### Color variation (ΔE_00_)

**Fig. 3:**
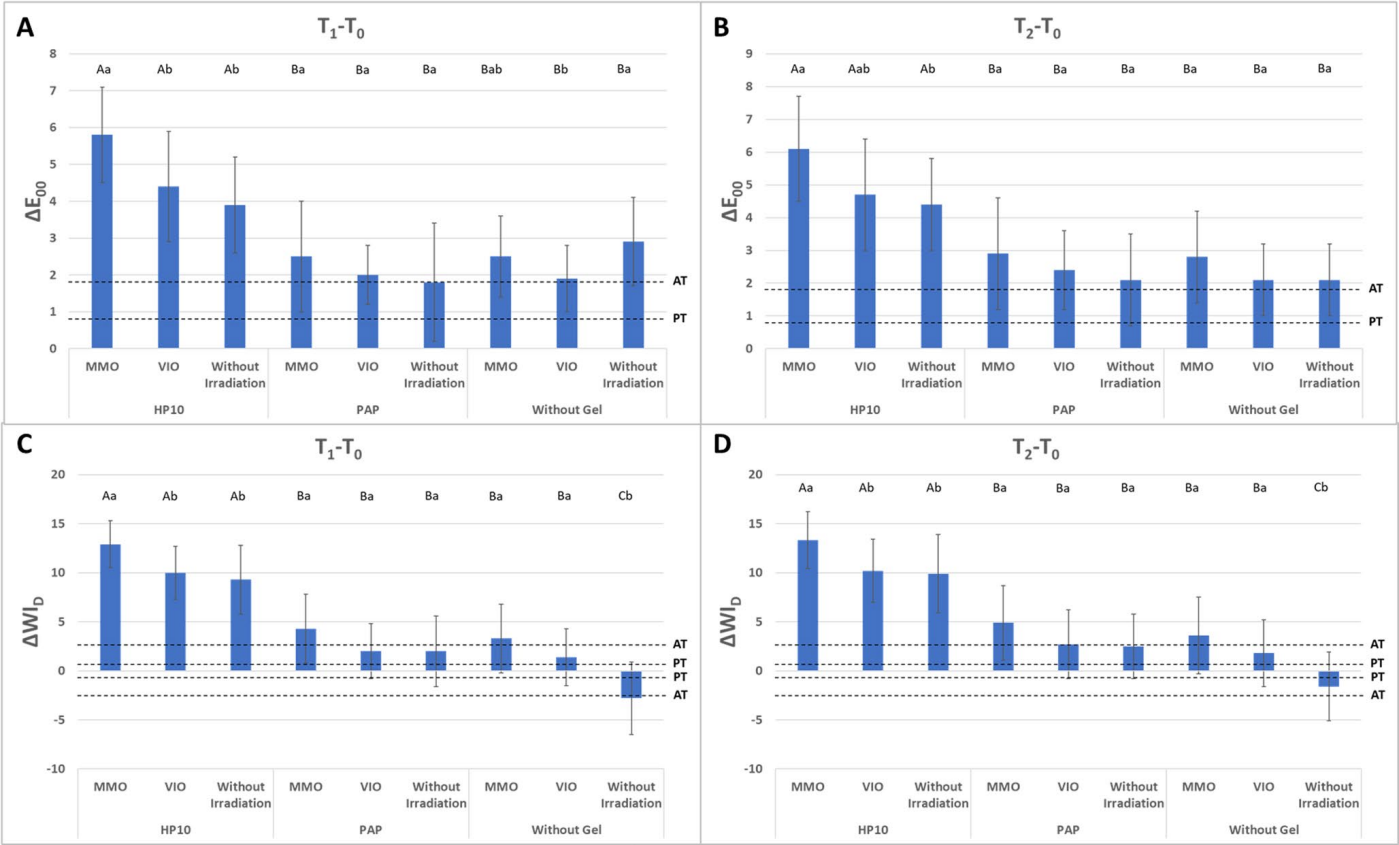
Means and standard deviations of ∆E_00_ and ∆WI_D_ according to the evaluated time points. Differences between letters (uppercase letters indicate different products irradiated by the same light source; lowercase letters indicate comparisons of the same product under different light sources) represent statistically significant differences (*p* ≤ 0.05), according to ANOVA and Tukey’s post hoc test (*p* < 0.05). For ∆E_00_ the PT and AT lines represent the perceptibility (0.8) and acceptability (1.8) thresholds, respectively. As for ∆WI_D_, PT and AT lines represent the perceptibility (0.72) and acceptability (2.62) thresholds, respectively

Figure 3A shows the ΔE_00_ values at T_1_ - T_0_ interval. A consistent pattern can be observed in which the groups treated with HP10 exhibited greater ΔE_00_ than those treated with PAP (*p* < 0.05). In turn, the PAP-treated groups showed no statistical difference than groups with no bleaching agent’s application (*p* > 0.05). This pattern was evident regardless of whether the treatment was combined with LED irradiation.

The combination of MMO with HP10 resulted in a greater ΔE_00_ compared to either VIO or no irradiation (*p* < 0.05), the latter two with no statistical difference (*p* > 0.05). For the PAP-treated groups, no statistically significant differences were observed between those with LED irradiation and those without (*p* > 0.05). For the groups treated solely with irradiation, VIO differed statistically from the non-irradiated group, producing the least color change (*p* < 0.05). The MMO-only group showed no statistical difference (*p* > 0.05) from the VIO and control groups.

Fourteen days elapsed from bleaching (T_2_ - T_0,_ Fig. 3B), the same behavior of the gels was observed: groups treated with HP10 exhibited greater ΔE_00_ than from those treated with PAP or without gel (*p* < 0.05). Focusing on LED-specific effects, within the HP10-treated groups, MMO greater than the no-irradiation group (*p* < 0.05), while VIO had no statistical differences to both. In the PAP-treated or no-gel groups, no statistical differences were observed between MMO, VIO, or no irradiation (*p* > 0.05).

### Whiteness index (ΔWI_D_)

The ΔWI_D_ results is displayed in Fig. 3C. Observing T_1_ and T_2_, the same pattern is evident, in which HP10 shows a statistically higher ΔWI_D_ than PAP and the groups without gel application (*p* < 0.05). However, all groups demonstrated higher ΔWI_D_ than NC group, which exhibited a slight darkening (*p* < 0.05). LED irradiation was capable of promoting a whitening effect, and no differences were noted between MMO and VIO (*p* > 0.05); and both exhibited higher ΔWI_D_ than the NC-group (*p* < 0.05). No differences in ΔWI_D_ were noted for PAP groups, regardless of the device or the absence light irradiation. Besides, PAP-treated groups exhibited lower ΔWI_D_ than HP10 groups. In the HP10-treated groups, MMO promoted higher ΔWI_D_ than VIO and to the non-irradiated group, which were statistically similar to each other (*p* > 0.05).

Fourteen days elapsed from bleaching (Fig. 3D), groups treated with HP10 exhibited higher ΔWI_D_ than those treated with PAP or without gel (*p* < 0.05). A slight darkening was noted for the untreated NC group, which differed from all other groups (*p* < 0.05). No differences were noted between PAP and groups treated only with light irradiation (*p* > 0.05) and no differences were noted between MMO and VIO (*p* > 0.05), although both showed higher ΔWI_D_ than the untreated C-group (*p* < 0.05). In the PAP-treated groups, neither the presence nor the absence of irradiation promoted statistical differences (*p* > 0.05), while HP10/MMO exhibited higher ΔWI_D_ than HP10/VIO (*p* < 0.05).

### Microhardness analysis

**Fig. 4:**
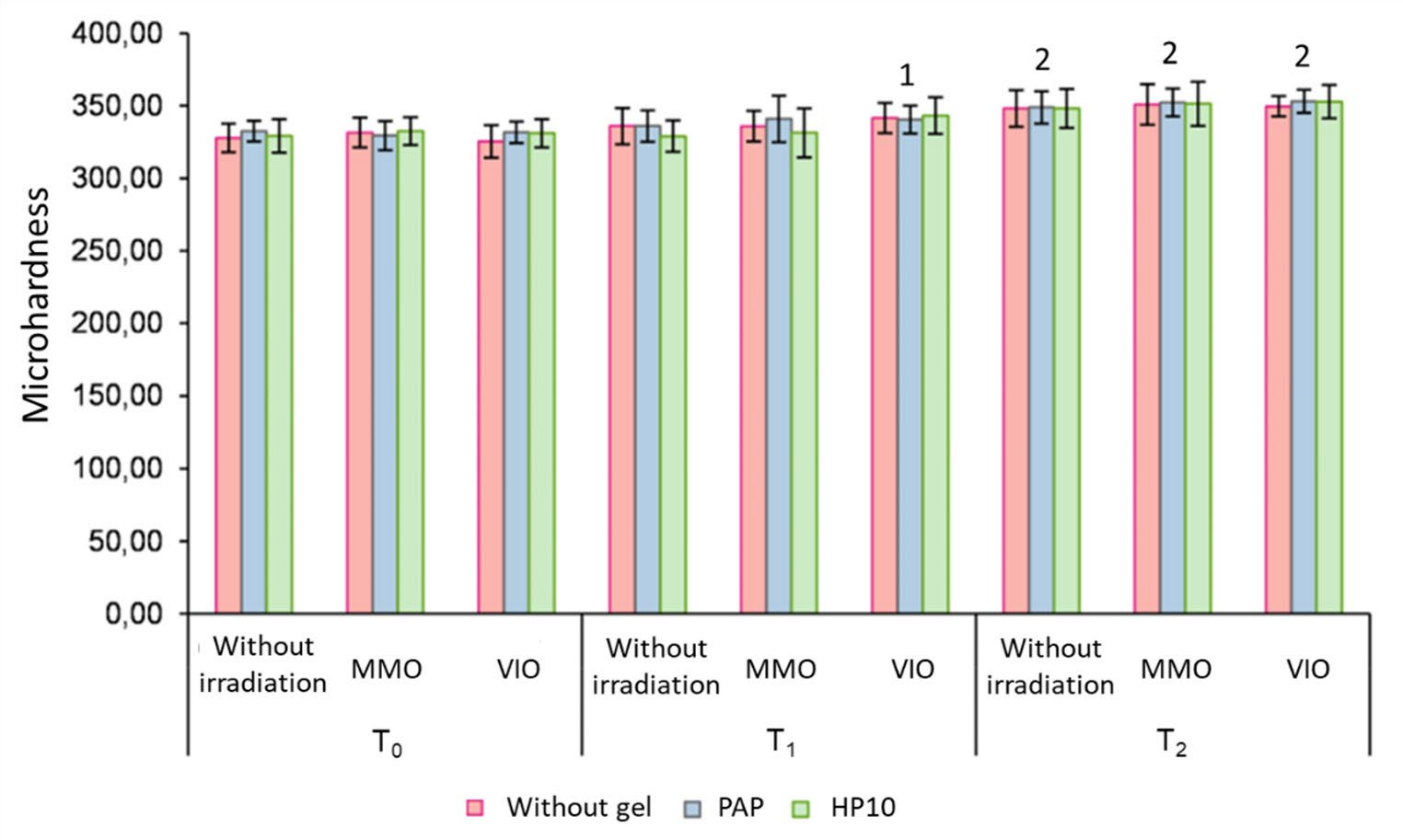
Means and standard deviations of Microhardness as a function of gel, light, and time. Linear mixed model for repeated measures over time and Tukey-Kramer test. p(gel) = 0.5587; p(light) = 0.2010; p(gel / light) = 0.8328; p(time) < 0.0001; p(gel / time) = 0.3093; p(light / time) = 0.0376; p(gel / light / time) = 0.5003. 1 Regardless of the gel, differs from the time point after pigmentation (T_0_), under the same light conditions (*p* ≤ 0.05). 2 Regardless of the gel, differs from the time points after pigmentation (T0) and 24 h after the last session (T1), under the same light conditions (p ≤ 0.05)

Figure 4 displays the enamel mean surface microhardness (SH). SH differences were noted at T_1_ and T_0_ only in the groups combined with VIO LED. All groups exhibited an increase in microhardness from T_1_ to T_2_ (*p* < 0.05). However, no significant differences were observed among treatments (*p* > 0.05)

### Scanning electron microscopy (SEM)

**Fig. 5:**
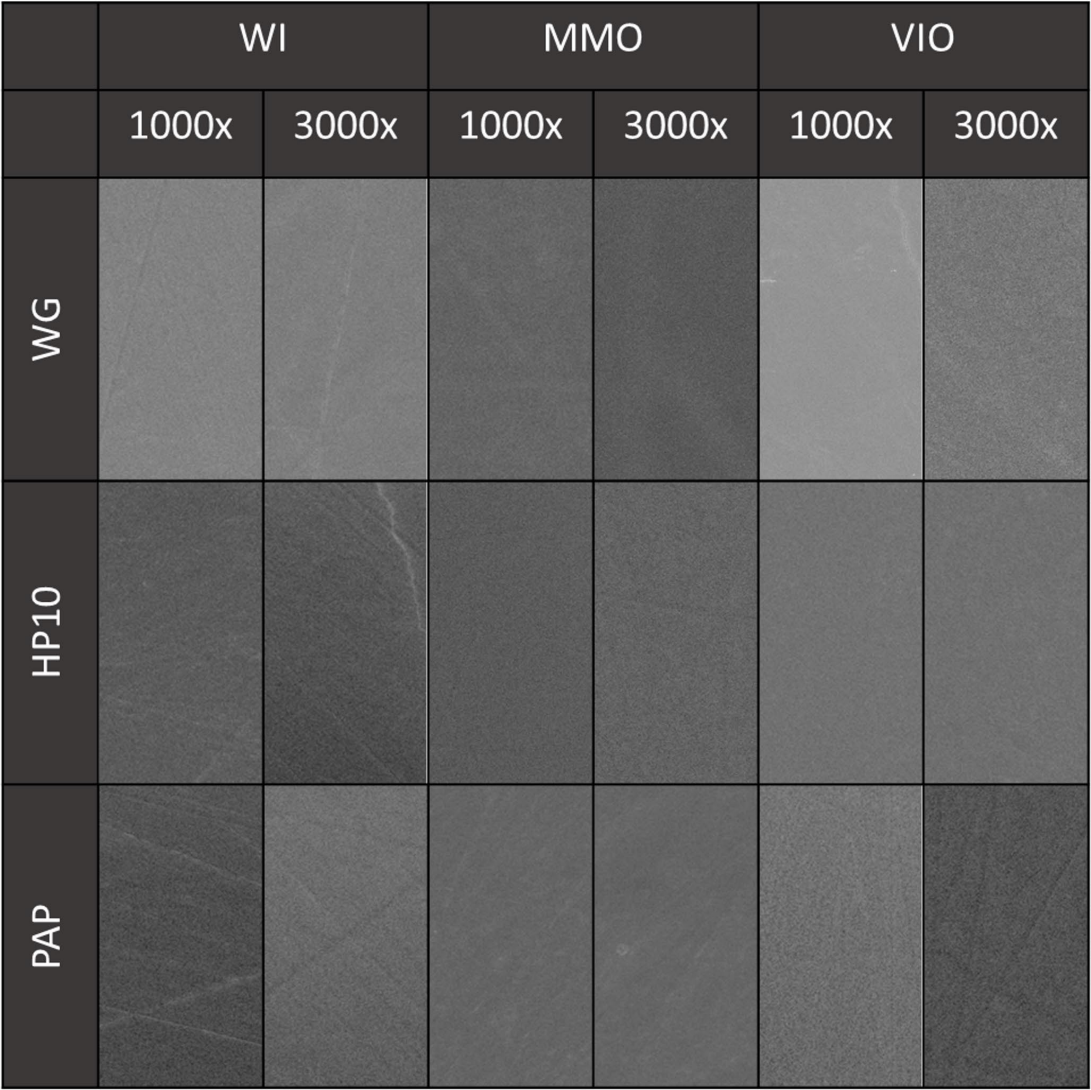
Qualitative SEM images of the enamel surface at 1000× and 3000× magnifications corresponding to the treatments. WG = Without Gel; WI = Without Irradiation

In the qualitative surface morphology images compiled in Fig. 5, no significant differences were observed among the groups, regardless of magnification. All specimens exhibited similar characteristics, showing a regular and flat surface without evidence of depressions or demineralization zones.

## Discussion

The first null hypothesis was rejected, as the results showed clear differences in irradiation stability and power output. The MMO device demonstrated superior performance, maintaining a constant average power of 250 mW/cm², whereas VIO exhibited a lower initial power (25 mW/cm²) that decreased to 15 mW/cm² after four minutes. The literature already describes MMO as an effective enhancer for low-concentration peroxide gels [3, 12], with favorable clinical outcomes even when used alone [5]. In the present study, this trend was corroborated by the association between MMO and HP10, which produced the highest levels of color alteration, in contrast to VIO, whose association did not yield any additional significant effect. The characterization of the LED devices also revealed a significant influence of physical barriers on emitted power. In the VIO device, the silicone barrier reduced the initial power by approximately 21%, stabilizing after four minutes, likely due to the gradual intrinsic power decay of the device itself. In the case of MMO, the acrylic tip caused a consistent average power loss of 32% throughout the irradiation period. These findings reinforce that the presence of physical barriers, although essential for containing the gel in VIO or for light transmission in MMO, may compromise device effectiveness. This aspect is rarely explored in the literature on light-assisted dental bleaching, and such data may provide valuable insights for the development of new, more efficient, and portable devices. The irradiance spectrum revealed distinct peaks for each device: 404 nm for MMO (violet) and 457 nm for VIO (blueish). Although evidence suggests that violet wavelength range is more effective in breaking down chromophores, the literature on this topic remains controversial and is more focused on violet than on blue wavelengths [25–27]. The lower power demonstrated by VIO throughout the study may partially explain, rather than wavelength itself, the higher effectiveness observed for MMO.

VIO is primarily an at-home approach that also functions as a mouth tray for gel application, and MMO is a well-documented professional clinical device for light-assisted bleaching or even light-only protocols [3, 5]. It would be expected that light activation could promote some degree of enhancement in the performance of the bleaching agent in direct contact with the tooth surface, independently of the treatment modality. However, such an effect was not observed for PAP. It may be argued that VIO provided benefits when associated with HP10, since a total exposure time of only 10 min per session resulted in bleaching efficacy comparable to that of the HP10-only group, which required 30 min of application (*p* > 0.05). This finding is particularly relevant given that MMO also failed to produce statistically significant improvements when combined with PAP. Nonetheless, MMO when associated with PAP or used alone, yielded results that exceeded the acceptability threshold, which is consistent with previous findings [5, 20]; the same was not observed for VIO. Importantly, MMO clearly enhanced the bleaching efficacy of HP10, although it was applied for a total of 20 min, compared with only 10 min for VIO.

The light-only and No treatment (NC) groups should not be overlooked, as these data suggest that the devices alone were capable of producing some degree of color change. While MMO consistently achieved values above the acceptability threshold, VIO maintained positive ΔWI_D_ values. In contrast, the NC group exhibited negative ΔWI_D_ values, which may be attributed to limitations inherent to the in vitro model. Overall, except for MMO, the observed variations were not clinically significant. These findings indicate that VIO does not provide intrinsic bleaching efficacy when used alone but may enhance the performance of HP10 through indirect mechanisms, such as thermal effects [12].

pH evaluation demonstrated that irradiation promoted a consistent reduction in final pH values, particularly with MMO. This finding aligns with previous reports indicating that acceleration of oxidative reactions leads to greater release of H⁺ ions, acidifying the medium [28]. Importantly, in none of the experimental groups did the pH values fall below the critical enamel dissolution threshold (5.5), suggesting that the protocols employed do not pose an immediate risk of demineralization.

The second null hypothesis was rejected, as colorimetric results revealed clear differences among the tested agents. PAP, either alone or combined with LEDs, did not produce statistically significant changes compared with the control groups, although a slight whitening trend was observed. In contrast, HP10 showed statistically significant differences at all time points, confirming its well-established bleaching potential [29]. The combination of HP10 with MMO yielded the highest color change values (ΔE_00_ and ΔWI_D_), reinforcing the synergistic effect previously reported in the literature [3, 12]. Interestingly, both MMO and VIO, even without gel application, produced slight but statistically significant differences compared with the non-irradiated control, suggesting that light exposure alone may play a role in color alteration. This observation is consistent with studies describing the direct photophysical action of specific wavelengths on organic pigments [3, 5].

The third null hypothesis was accepted, as microhardness analysis revealed a homogeneous behavior among the groups, with a gradual increase over time. This effect is attributed to continuous exposure to the remineralizing solution used in the experimental protocol. Qualitative SEM images corroborated these findings, showing intact enamel surfaces across all groups, including those treated with PAP and HP10. These results suggest that both agents are safe for hard dental tissues, in agreement with previous studies reporting no structural damage [16, 19, 30].

From a clinical perspective, the findings allow for several considerations. First, although PAP exhibited slight bleaching activity, its efficacy remains questionable compared with HP10. This scenario is true even when used under longer application times than those recommended by the manufacturer. This raises doubts about the true clinical applicability of PAP as an alternative to peroxides, at least in the adopted protocols, using the commercial product tested. Second, the combination of VIO with PAP did not produce any additional benefit, which may justify the recent commercial reformulation of the product by the manufacturer, now marketed in strips and pen formats without light-activation devices.

This study presents some important limitations. The primary limitation concerns the need to adjust application times across different protocols to ensure experimental comparability. This issue is intrinsically related to the manufacturers’ recommended application protocols. For PAP+ (HiSmile), the manufacturer states that a single to double application is sufficient to achieve the desired clinical bleaching outcome. However, additional sessions may be performed to maintain or further enhance bleaching effects, as per manufacturer’s directions. The kit provides a total of 6 PAP+ capsules. After determining the automatic shut-off time of the VIO device, a single session was defined as 10 min, with interrupted irradiation. The shortest at-home hydrogen peroxide protocol was then established according to the manufacturer’s instructions. A 10% hydrogen peroxide gel (FGM) was selected, consisting of daily applications of 30 min for 7 days. Subsequently, a gold-standard light-assisted bleaching protocol was required to allow comparison with the VIO device. As there are no well-documented devices designed for light-assisted bleaching in an at-home setting, the MMO device was selected due to its extensive and well-established supporting literature [3, 5, 12, 20, 31, 32]. The most appropriate protocol was chosen to represent an intermediate condition among PAP+, VIO, and HP10 protocols, resulting in a final regimen of 20 min of irradiation with 2-minute intervals.

Overall, prolonged application of PAP, exceeding the manufacturer’s recommendations by at least twofold, failed to achieve significant bleaching outcomes. In contrast, HP10 demonstrated satisfactory performance even with shorter exposure times. Notably, when HP10 was associated with VIO, the exposure time was reduced to 10 min while still achieving bleaching efficacy comparable to the 30-minute non-irradiated protocol. From this perspective, the adoption of different protocols may be interpreted not solely as a limitation, but as an approach that provides a broader and more informative evaluation of the materials and devices tested. It is well established that bleaching efficacy is highly dependent on the product formulation, concentration, and exposure time, with cumulative effects being widely documented [1, 2, 33, 34]. Higher-concentration gels tend to produce faster bleaching until a saturation plateau is reached, whereas lower-concentration gels can achieve similar final outcomes but require longer exposure times to reach this plateau. Based on the proposed mechanism of action of PAP, improved performance would be expected under more prolonged application protocols. Another limitation is the exclusive focus on hard dental tissues, with SEM analysis adopting a qualitative approach due to the restricted sample size (*n* = 3). The absence of an evaluation of effects on soft tissues and dental pulp precludes definitive conclusions regarding the clinical safety of PAP, an essential aspect considering that the product is applied directly onto gingival surfaces.

Future investigations should explore alternative, non-proprietary, PAP formulations, with different application times, and, most importantly, the response of soft and pulpal tissues to product exposure. Only then will it be possible to robustly determine whether PAP represents a viable alternative to traditional peroxide-based bleaching agents. Based on the above, it can be inferred that the short-term protocol is not suitable for the product and that no significant benefits were observed from LED association; however, a more prolonged protocol may justify PAP use, especially considering its safety for hard tissues.

## Conclusion

VIO exhibited significantly lower power and stability than MMO, but arguably, both enhanced the efficacy of HP10. Under the tested protocol, PAP+ failed to induce significant bleaching, regardless of association with LED irradiation, while HP10 effectively induced bleaching independently of LED use. Importantly, none of the tested treatments resulted in microhardness loss or morphological damage to the enamel surface.

## Data Availability

No datasets were generated or analysed during the current study.
